# Gastrointestinal manifestations in patients with gastric adenocarcinoma and proximal polyposis of the stomach (GAPPS): a systematic review with analysis of individual patient data

**DOI:** 10.1186/s13053-024-00284-6

**Published:** 2024-07-22

**Authors:** PA Skat-Rørdam, Y Kaya, N Qvist, TvO Hansen, TD Jensen, JG Karstensen, AM Jelsig

**Affiliations:** 1 Department of Clinical Genetics, Copenhagen University Hospital, Blegdamsvej 9, Copenhagen, Rigshospitalet, DK-2100 Denmark; 2 University of Southern Denmark, Odense, Denmark; 3 Research Unit for Surgery, Odense University Hospital, Odense, Denmark; 4 Dept. of Clinical Medicine, University of Copenhagen, Copenhagen, Denmark; 5 Dept. of Clinical Genetics, Vejle Hospital, Vejle, Denmark; 6 Danish Polyposis Registry, Gastrounit, Copenhagen University Hospital – Amager and Hvidovre, Hvidovre, Denmark

**Keywords:** Systematic review, Gastric adenocarcinoma and proximal polyposis of the stomach, Gapps, *APC*, Promotor region, Gastric polyps

## Abstract

**Background and aim:**

Gastric adenocarcinoma and proximal polyposis of the stomach (GAPPS) is an autosomal dominant syndrome characterized by fundic gland polyps (FGP) as well as an increased risk of gastric cancer. The syndrome has been recognized as a clinical entity for less than a decade. A clinical suspicion may be complex and can vary from incidental findings of FGPs at gastroscopy to obstructive symptoms with dyspepsia and vomiting. The diagnosis is established by genetic detection of a pathogenic variant in the promotor 1B region of the APC gene. As of yet there are no established clinical criteria for the diagnosis. To increase knowledge of the condition and to discuss possible genetic testing and surveillance strategies, we performed a systematic review of all reported patients with GAPPS.

**Methods:**

This review was organized according to PRISMA guidelines. The search, which was conducted on September 7th, 2023, was applied to MEDLINE and restricted to only humans and papers in the English language. Only the studies on patients/families with GAPPS verified by identification of a pathogenic variant in the *APC* promoter 1B were included.

**Results:**

Twelve publications with a total of 113 patients were identified. In all instances the diagnosis was genetically verified with reports of four different variants within the *APC* promotor 1B region. Eighty-eight patients (90.1%) had gastric polyps, of these seven patients had low-grade dysplasia and five patients had both low- and high-grade dysplasia. Thirty-seven patients (45.7%) underwent gastrectomy. There were no reports of duodenal polyps (0%). Gastric cancer was found in 31 patients (30.1%) with a median age of 48 years (range 19–75). Twenty-six patients died (23.2%) of which 19 had developed gastric cancer (73.1%). One patient was diagnosed with metastatic colorectal cancer (2.2%) and died at 73 years of age. Nineteen patients had colorectal manifestations with < 20 polyps (41.3%).

**Conclusion:**

Patients with a pathogenic variant in the *APC* promoter 1B region have an increased risk of gastric polyposis and early-onset gastric cancer. However, there is considerable variation in clinical expression and penetrance, which makes decisions on surveillance and the timing of prophylactic gastrectomy challenging.

**Supplementary Information:**

The online version contains supplementary material available at 10.1186/s13053-024-00284-6.

## Background

Gastric fundic gland polyps (FGP) are most often sporadic and an incidental finding at gastroscopy. In familial adenomatous polyposis (FAP) the development of multiple gastric polyps are common [1], but the malignant potential is considered low [2]. Gastric adenocarcinoma and proximal polyposis of the stomach (GAPPS) is an autosomal dominantly inherited syndrome which is also characterized by fundic gland polyps and gastric cancer. The syndrome was originally described by *Worthley* et al. in 2012 [3]. The underlying genetic cause of GAPPS is heterozygous pathogenic single nucleotide variants in the promoter 1B region of the APC gene, whereas large deletions of the promotor 1B region predisposes to FAP. The single nucleotide variants in the promotor 1B of the APC gene interrupt the Ying Yang 1 (YY1) binding site, reducing the expression from promoter 1B [4]. GAPPS presents with multiple polyps in the gastric body and fundus but sparing the antrum. FAP can also present with FGPs, but the phenotypic difference lies in the colonic and duodenal manifestations [2, 5]. FAP is characterized by hundreds to thousands of colorectal polyps and a cumulative incidence of duodenal polyps of 90%, while colonic- and duodenal polyposis is typically not seen in patients with GAPPS [2–8].

Still little is known about the penetrance of gastric cancer in GAPPS but it is believed to be high [3, 9]. Due to this risk patients with GAPPS are observed with routine gastroscopies and polypectomies. Prophylactic gastrectomy is also performed usually due to the finding of dysplasia in the FGPs, severe polyposis or gastric cancer. However, there are no established clinical guidelines.

Since the discovery of the syndrome, clinical diagnostic criteria have been proposed. *Worthley* et al. recommend the following diagnostic criteria for GAPPS (all must be fulfilled):

(1) gastric polyps restricted to the body and fundus with no evidence of colorectal or duodenal polyposis; (2) > 100 polyps carpeting the proximal stomach in the index case or > 30 polyps in a first-degree relative of another case; (3) predominantly FGPs, some having regions of dysplasia (or a family member with either dysplastic FGPs or gastric adenocarcinoma); (4) an autosomal dominant pattern of inheritance. Exclusions include other gastric polyposis syndromes and the use of proton pump inhibitors (PPIs) [3]. However, genetic analysis is now used to confirm a clinical suspicion of GAPPS.

In this study, we performed a systematic review of reported patients with GAPPS to explore the recommendations for genetic testing, future surveillance, and prophylactic gastrectomy in patients with a pathogenic variant in the *APC* promoter 1B.

## Methods

This review was performed according to PRISMA guidelines and using the COVIDENCE^®^ online program. The literature search was restricted to MEDLINE (PubMed) database with the following criteria: *(gapps OR gastric adenocarcinoma and proximal polyposis of the stomach OR gastric polyps) AND (apc OR (autosomal dominant) OR (promoter 1B)* and restricted to only humans and articles in the English language. The search was conducted on September 7th, 2023. Only original studies including patients/families with a pathogenic variant in the *APC* promoter 1B were included. Patients with FAP, Peutz-Jeghers syndrome (PJS), Juvenile polyposis syndrome (JPS), and other hereditary gastric syndromes such as Lynch syndrome (LS) and hereditary diffuse gastric cancer (HDGC) were excluded.

## Results

**Fig. 1:**
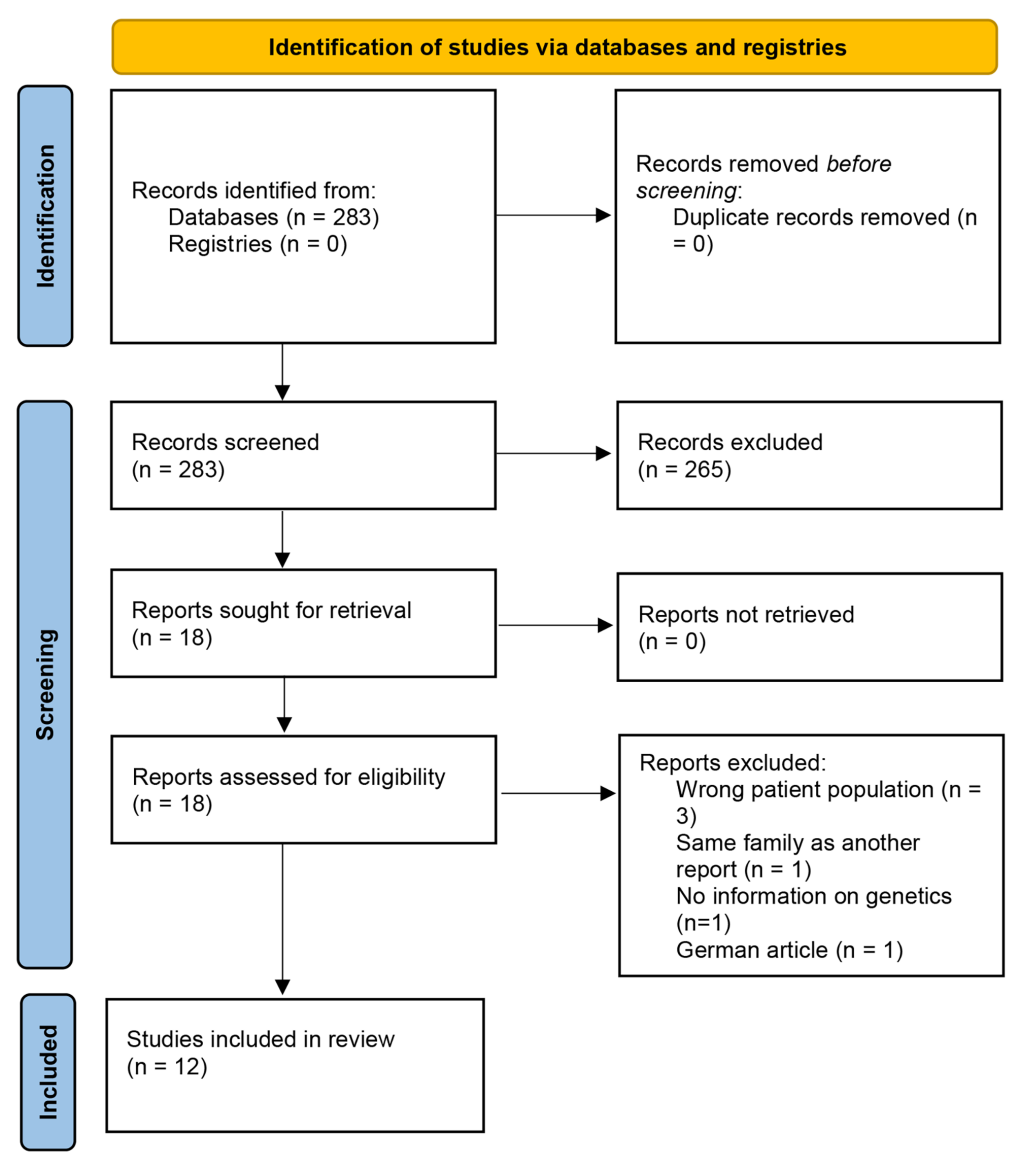
PRISMA flowchart

A total of 283 articles were identified, and 12 articles were included (Fig. 1) with a total of 113 patients (Table 1) from 27 different families [3, 4, 6, 7, 9–16]. The age at diagnosis was reported in 82 patients with a mean age of 43 years, ranging from 10 to 92 years. All patients (supplemental Table 1) met the criteria of GAPPS either after the confirmation of a pathogenic variant in the *APC* promotor 1B (98 patients) or by being an obligate carrier of a gene variant (15 patients). The patients were from Asia, Europe, Australia, and North America.

**Table 1:** Summary of patient characteristics

Parameter	*n*	%
Number of patients	113	
Number of families	27	
Mean age of diagnosis (years)	43 (*n* = 82)	
Age range (years)	10–92	
Gender M/F	18/36	33%/66%
Obligate carriers	15	13%
Reported pathogenic variant Pathogenic variants	98	77.8%
- *APC* variant: c.-191T > C	52	53.1%
- *APC* variant: c.-195 A > C and c.-125delA	33	33.7%
- *APC* variant: c.-192 A > G	2	2%
- *APC* variant: c-191T > G	1	1%
- Unspecified	10	10.2%
Gastric polyposis	88 (*n* = 97)	90.1%
Low-grade dysplasia/low- and high-grade dysplasia/ unspecified	7/5/5 (*n* = 24)	29.2%/ 20.8%/ 20.8%
Duodenal polyps	0 (*n* = 83)	0%
Colorectal polyps	19 (*n* = 46)	41.3%
Gastric cancer diagnosis	31 (*n* = 103)	30.1%
- Adenocarcinoma	22	70.9%
- Unknown histopathology	9	16.1%
- Median age (years)	48 (*n* = 25)	
- Age range (years)	19–75	
- Gender M/F	5/17	22.7%/ 77.3%
Colorectal cancer	1 (*n* = 46)	2.2%
Mortality	26 (*n* = 112)	23.2%
- Death from gastric caner	19	73.1%
- Death from colorectal cancer	1	3.8%
- Unspecified	6	23.1%
- Median age (years)	52 (*n* = 12)	
Gastrectomy	37 (*n* = 81)	45.7%
- Median age (years)	37 (*n* = 15)	
- Age range (years)	19–66	

The figures in brackets denote the number of patients where there was a specific information on the event

### Genetics

**Fig. 2:**
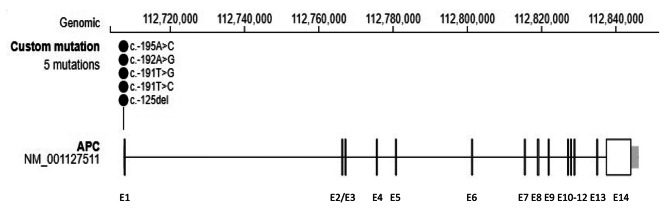
Lollipop plot showing the APC NM_001127511.3 transcript including promoter 1B (exon 1), as well as identified promoter variants (c.-125del, c.-191T > C, c.-191T > G, c.-192A > G and c.-195A > C) described in relation to gastric adenocarcinoma and proximal polyposis of the stomach (GAPPS). Eighteen families had the promotor variant c.-191T > C, one family had the promotor variant c.-192A > G, one family had the promotor variant c.-191T > G, one family had only the promotor variant c.-195A > C and one family had the promotor variants c.-195A > C and c.-125delA. E1-E14 indicates exon numbering.

Four different variants were reported within the *APC* promotor 1B region in the 27 families (Table 1, supplemental table 1 and Fig. 2), the most common being c.-191T > C which was found in 52 patients. Thirty-three patients were reported having two *APC* promotor 1B variants: c.−195 A > C and c.−125delA. Two patients had the c.-192 A > G variant, while one had the c.-191T > G variant. Ten patients were reported to have a variant in the promoter 1B of the APC gene but with no specification of where the variant was localized. Fifteen patients were identified as obligate carriers.

### Polyps

**Fig. 3:**
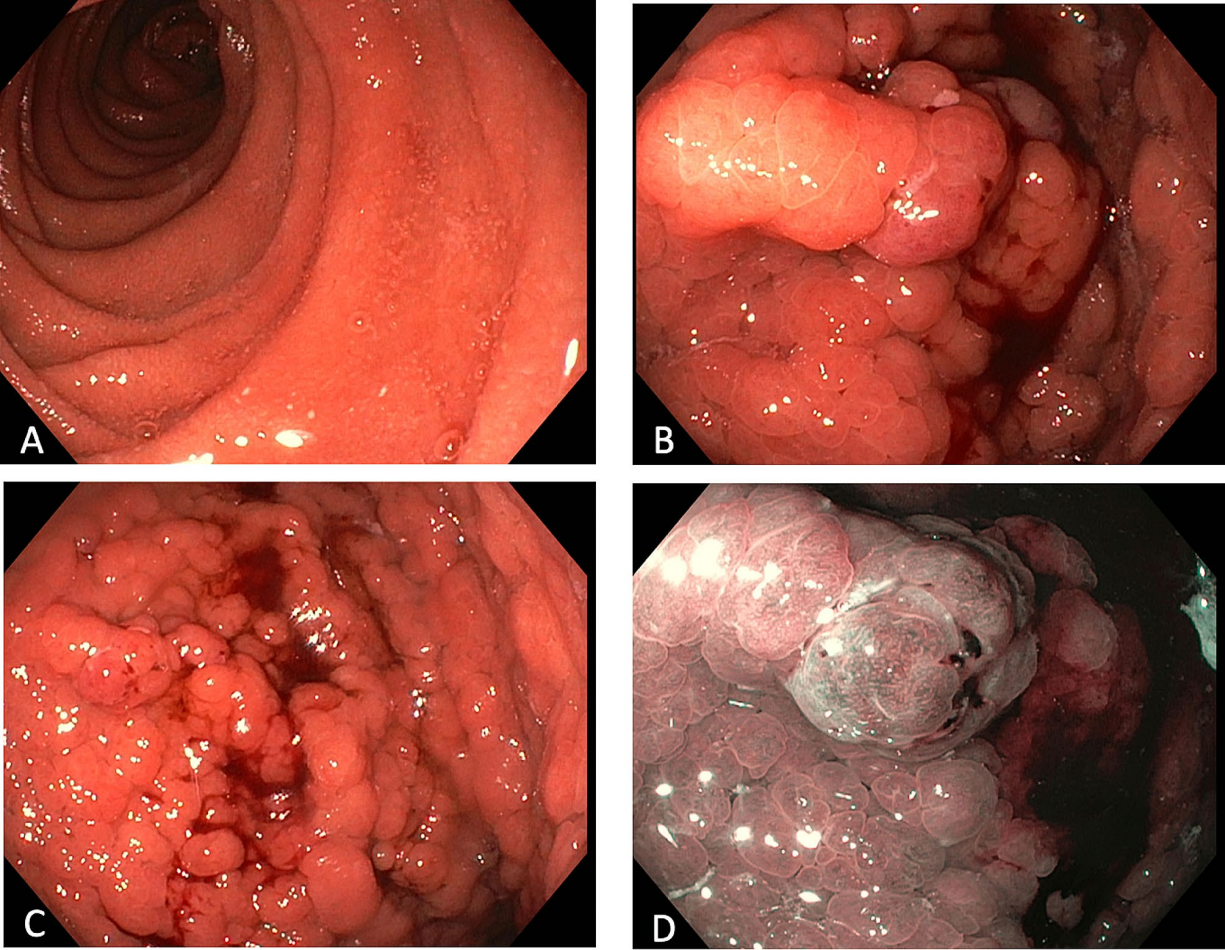
Endoscopic images from a 25-year-old GAPPS patient seen at Hvidovre Hospital, Denmark. **(a)** Normal mucosa in the duodenum without any adenomas, **(b)** and **(c)** extensive gastric carpeting of fundic gland polyps, some with low-grade dysplasia, and **(d)** narrow-band imaging of the identical polyps

Eighty-eight patients (90.1%) had gastric fundus polyps that were histopathologically described as gland polyposis (Fig. 3). Nine patients (9.3%) had no FGPs. Dysplasia status of the FGPs were described in 24 patients [3, 4, 6, 9, 11–16]. Seven patients had low-grade dysplasia, three patients had both low- and high-grade dysplasia, two patients had low-grade dysplasia which progressed to high-grade dysplasia, five patients had unspecified dysplasia while the remaining seven patients had no dysplasia.

Information regarding duodenal polyposis was available in 17 families (63%) and was absent in all.

In total, 19 out of 46 examined patients had colorectal manifestations (41.3%). They presented with less than 20 either colorectal hyperplastic polyps, simple tubular adenomas or tubulovillous adenomas.

### Cancer

The information on development of gastric cancer was available in 103 patients and was reported in 31 patients (30.1%) with a histologically proven adenocarcinoma, including one case of carcinoma in situ, in 22 patients (70.9%), and no information on the histopathology in the remaining nine patients (Table 1). The information on gastrectomy was available on 25 of the 31 patients with gastric cancer and was performed on nine of those patients (36%). The median age at cancer diagnosis was 48 years (mean age 44 years), ranging between 19 and 75 years. One patient (2.2%) was diagnosed with metastatic colorectal cancer and died at the age of 73.

### Mortality

Twenty-six patients died (23.2%), of which 19 had developed gastric cancer (73.1%) and one had developed metastatic colorectal cancer (3.8%). Among the remaining six patients, there was no information on the cause of death (Table 1).

### Gastrectomy

Information regarding gastrectomy was available on 81 patients – and was performed in 37 patients (45.7%) (Table 1). Of these, five underwent gastrectomy due to gastric cancer (13.5%). Two of these patients were observed with gastroscopy every three months for 12 months prior to the diagnosis of gastric cancer. Four were found to have an incidental gastric adenocarcinoma (10.8%), of which two had high-grade dysplasia focally before gastrectomy, one had low-grade dysplasia and one had an unknown dysplasia status. Gastric polyposis was found in the remaining 28 cases (75.7%), with reports of low-grade dysplasia in four patients, two reports of focal dysplasia, one report of dysplasia without specification and one report of a patient with both low-grade and high-grade dysplasia. In the remaining 20 cases there were no reports of dysplasia status, yet gastrectomy was performed as a prophylactic measure due to massive polyposis and/or a family history of gastric cancer. No postoperative mortality was reported. The median age at gastrectomy was 37 years (mean age 41 years), ranging from 19 to 66.

## Discussion

This review covers 12 case studies with 113 patients diagnosed with a pathogenic variant in the promoter 1B region of the APC gene. Eighty-eight (90.1%) patients had gastric polyps, 37 (45.7%) underwent gastrectomy, and gastric cancer was detected in 31 patients (30.1%) with the youngest patient being diagnosed at 19 years of age. Thus, based on our collected data, GAPPS is a syndrome with a substantial risk of developing gastric cancer from an early age; however, there is a considerable variability in expression within and between families. Furthermore, penetrance could be reduced which is not necessarily reflected in our data due to ascertainment bias. Only a few other hereditary syndromes increase the risk of gastric cancer to such an extent with HDGC being the one with the highest penetrance. Studies suggest that HDGC is associated with a gastric cancer risk of up to 42% [17, 18]. LS, JPS, and PJS are other syndromes that increase the risk of gastric cancer with studies reporting a gastric cancer risk of between 2 and 8%, 5–21% and 24%, respectively [19–23].

Globally, gastric cancer is considered the fifth most common cancer and it is the fourth most common cause of cancer death and thus there is a motivation to identify at-risk patients and offer surveillance [24]. However, in genetic syndromes that predispose to gastric cancer, surveillance is not always straightforward as changes in the gastric mucosa are hard to detect with routine gastroscopy. The European Hereditary Tumour Group (EHTG) and the American College og Gastroenterology (ACG) have established clinical guidelines for hereditary gastrointestinal cancer syndromes such as PJS and JPS, however not including GAPPS [25, 26]. Patients with PJS are often recommended a baseline examination for gastric cancer starting at the age of eight and repeated at the age of 18 years if no polyps are found and from then every three years. Concerning JPS, surveillance often starts at 12–15 years of age and is repeated every 1–3 years depending on the severity of polyposis, though recent guidelines differentiate between the genetic subtypes as patients with a pathogenic variant in *SMAD4* have a more severe gastric phenotype. In these patients, gastrectomy is often performed because of severe gastric polyposis with obstructive symptoms and their risk of gastric cancer has been reported to be roughly 5% [27].

Similar clinical guidelines could be proposed for GAPPS, even though risk estimates for cancer development are uncertain. Also, the difficulty with surveillance of FGPs in patients with GAPPS is collecting the right sample biopsies reflecting of the underlying status of dysplasia. This becomes more problematic in patients with > 100 larger FGPs in the gastric area mixed with adenomatous polyps, which increases the risk of potentially missing areas of malignant transformation and focal progression. For example, one patient in this study was suspected of gastric cancer due to histopathological findings of foveolar-type dysplasia/adenoma or adenocarcinoma with low-grade atypia. However, histopathology on the following specimens from the gastrectomy showed only FGPs with dysplasia, thus illustrating the difficulty in preoperative diagnostics of gastric cancer [16]. Furthermore, we also found that there is a remarkable variation in clinical expression in patients with GAPPS. The youngest patient to be diagnosed with gastric adenocarcinoma in our review was 19, while the oldest was 75. The decision of when prophylactic gastrectomy is indicated can be challenging, and various variables should be taken into consideration including the risk of serious complications such as anastomotic leakage. In addition, there is still an increased morbidity and mortality risk post-gastrectomy along with other long-term complications such as diarrhea, dumping syndrome, malnutrition and more. Yet, long-term gastroscopy surveillance does not seem favorable as there are reports of interval cancers [9, 28]. It therefore seems that prophylactic gastrectomy should be recommended in patients with GAPPS if they have clinical manifestations e.g., polyposis with dysplasia. However, one should keep in mind, that we do not know the natural history. Also, GAPPS could be underdiagnosed, and the current results may be overestimating the risk of severe disease. The increased use of genetics in diagnostics today, will hopefully lead to more knowledge on the phenotypic spectrum. In this review we found that not all patients who were diagnosed with gastric cancer underwent gastrectomy. In the observed cases, the lack of gastrectomy was either due to advanced metastatic disease or death shortly after diagnosis.

The Danish Surgical Society and the Danish Society of Medical Genetics have developed a guideline for GAPPS [29]. They recommend upper endoscopic surveillance annually in patients from the age of 15 and eventually more frequently if polyps are present. Prophylactic gastrectomy is recommended if dysplasia is identified in the biopsy specimen. All patients should be followed afterwards upon duodenal manifestations according to the Spiegelman stage of duodenal polyps. However, these recommendations are based on expert opinions and as this study shows, there seems to be little or no risk of duodenal neoplasia.

Genetic analysis is a cornerstone in the diagnosis of GAPPS. Although clinical diagnostic criteria have been proposed, it is difficult to differentiate between GAPPS and patients with sporadic fundic gland polyps or gastric polyps for other reasons. Consequently, it is also difficult to recommend criteria for when to perform genetic testing upon detection of such polyps. In general, a family history of FAP or colonic FAP manifestations with several adenomas and/or extraintestinal FAP manifestations should lead to a suspicion of FAP. The detection of hamartomatous juvenile polyps along with an inflammatory, edematous, and erythematous appearance of the gastric mucosa should lead to a suspicion of *SMAD4-*related JPS [27]. Gastric polyps are also seen in *PTEN* hamartoma tumor syndrome (Cowden syndrome), but these are often hamartomas, ganglioneuromas and hyperplastic polyps, and the patients may experience other symptoms such as dermatological manifestations and macrocephaly [30, 31]. Glycogenic acanthosis of the esophagus is also reported in up to 80% of these patients [32]. In addition, FGPs are also seen sporadically and associated with the use of PPIs. One study found FGPs in 23% of patients taking PPIs, compared with a 12% incidence in patients not taking PPIs [33]. The malignant potential of sporadic fundic gland polyps seems to be very low, as is the prevalence of *H. pylori* infection [34]. In general, early gastric cancer (under 40–50 years of age) with or without polyps should lead to genetic testing, also to rule out other genetic causes to early gastric cancer. We also propose that one should consider GAPPS in the case of unexplained corpus fundic polyposis in addition to a family history of gastric cancer – no matter the age at gastric cancer. This is also reflected in our results as the age of gastric cancer varied (from 19 to 75 years of age).

Today, most genetic analyses are done with a next generation sequencing (NGS) panel comprising several polyposis-associated genes (including *SMAD4* and *APC*). However, it is worth noting that the promoter 1B region of *APC* is not always included and additional genetic analyses, including whole genome sequencing (WGS), could be used to detect variants in this region of the APC gene. The youngest person to be diagnosed with GAPPS in this review was 10 years old and the youngest person to be diagnosed with gastric cancer was 19 years old. To date gastric cancer in GAPPS has not been reported in childhood and this raises the question of whether minors should be genetically tested for GAPPS. In all circumstances, patients diagnosed with or suspected of GAPPS should be referred to genetic counselling.

Although pathogenic variants are detected in the promotor 1B region of *APC*, there is, based on this review, nothing that suggest that these patients have other manifestations of FAP, including both intestinal and extraintestinal manifestations. Nineteen patients out of 46 examined (41.3%) presented with colorectal manifestations, each in the form of a scarce amount of either hyperplastic polyps, simple tubular adenomas, or tubulovillous adenomas in the colorectal system. None had a colorectal phenotype resembling FAP. The high percentage of colorectal polyps may well represent the high prevalence of sporadic colorectal polyps that is also seen in the background population [35]. Only one patient at the age of 73 was diagnosed with colorectal cancer [7]. Thus, now there is no evidence to suggest that patients should be offered follow-up colonoscopy albeit one can choose to perform a base-line colonoscopy upon diagnosis.

The limitation of this review is that several case reports did not present information on all included variables. This resulted in multiple patients with either unknown individual characteristics and/or endoscopic findings, making it difficult to make a comprehensive descriptive analysis. Furthermore, the results on cancer and polyposis risk are probably affected by ascertainment bias.

## Conclusion

We observed a noticeably increased risk of gastric polyposis and early-onset gastric cancer in the included GAPPS patients with a remarkable variation in clinical expression but an overall low prevalence of colorectal adenomatosis and colorectal cancer. This suggests that surveillance of the upper gastrointestinal tract is warranted, but that colonoscopy could be omitted.

### Electronic supplementary material

Below is the link to the electronic supplementary material.

Supplementary Material 1

## Data Availability

No datasets were generated or analysed during the current study.

## Declarations

### Guarantor of the article

PASK and YK had full responsibility for the conduct of the review and have control of the decision to publish.

## Abbreviations

GAPPS: Gastric adenocarcinoma and proximal polyposis of the stomach
FGP: Fundic gland polyps
FAP: Familial adenomatous polyposis
YY1: Ying Yang 1
PPI: Proton pump inhibitor
PJS: Peutz–Jeghers syndrome
JPS: Juvenile polyposis syndrome
LS: Lynch syndrome
HDGC: Hereditary diffuse gastric cancer
EHTG: European hereditary tumour group
ACG: American college of gastroenterology
NGS: Next–generation sequencing
WGS: Whole genome sequencing

## References

[1] Spiegel A, Stein P, Patel M, Patel R, Lebovics E. A report of gastric fundic gland polyps. Gastroenterol Hepatol (N Y). 2010;6(1):45–8. 20567540 PMC2886442

[2] Karstensen JG, Bülow S, Højen H, Jelsig AM, Jespersen N, Andersen KK, Wewer MD, Burisch J, Pommergaard HC. Cancer in patients with familial adenomatous polyposis: a nationwide Danish cohort study with matched controls. Gastroenterology. 2023;165(3):573–e5813. 10.1053/j.gastro.2023.05.010. 37201686 10.1053/j.gastro.2023.05.010

[3] Worthley DL, Phillips KD, Wayte N, Schrader KA, Healey S, Kaurah P, et al. Gastric adenocarcinoma and proximal polyposis of the stomach (GAPPS): a new autosomal dominant syndrome. Gut. 2012;61(5):774–9. 10.1136/gutjnl-2011-300348. 21813476 10.1136/gutjnl-2011-300348

[4] Li J, Woods SL, Healey S, Beesley J, Chen X, Lee JS, et al. Point mutations in exon 1B of APC reveal gastric adenocarcinoma and proximal polyposis of the stomach as a familial adenomatous polyposis variant. Am J Hum Genet. 2016;98(5):830–42. 10.1016/j.ajhg.2016.03.001. 27087319 10.1016/j.ajhg.2016.03.001 PMC4863475

[5] Karstensen JG, Burisch J, Pommergaard HC, Aalling L, Højen H, Jespersen N, Schmidt PN, Bülow S. Colorectal Cancer in individuals with familial adenomatous polyposis, based on analysis of the Danish polyposis Registry. Clin Gastroenterol Hepatol. 2019;17(11):2294–e23001. 10.1016/j.cgh.2019.02.008. 30743005 10.1016/j.cgh.2019.02.008

[6] Kunovsky L, Kala Z, Potrusil M, Novotny I, Kubes V, Prochazka V. A central European family with gastric adenocarcinoma and proximal polyposis of the stomach. Gastrointest Endosc. 2019;90(3):523–5. 10.1016/j.gie.2019.05.001. 31077701 10.1016/j.gie.2019.05.001

[7] Foretová L, Navrátilová M, Svoboda M, Grell P, Nemec L, Sirotek L, et al. GAPPS - gastric adenocarcinoma and proximal polyposis of the stomach syndrome in 8 families tested at Masaryk Memorial Cancer Institute - Prevention and Prophylactic Gastrectomies. Klin Onkol. 2019 Summer;32(Supplementum2):109–17. 10.14735/amko2019S109. English. 10.14735/amko2019S109 31409086

[8] Bülow S, Björk J, Christensen IJ, Fausa O, Järvinen H, Moesgaard F, Vasen HF, DAF Study Group. Duodenal adenomatosis in familial adenomatous polyposis. Gut. 2004;53(3):381–6. 10.1136/gut.2003.027771. 14960520 10.1136/gut.2003.027771 PMC1773976

[9] Repak R, Kohoutova D, Podhola M, Rejchrt S, Minarik M, Benesova L, Lesko M, Bures J. The first European family with gastric adenocarcinoma and proximal polyposis of the stomach: case report and review of the literature. Gastrointest Endosc. 2016;84(4):718–25. 10.1016/j.gie.2016.06.023. 27343414 10.1016/j.gie.2016.06.023

[10] Mitsui Y, Yokoyama R, Fujimoto S, Kagemoto K, Kitamura S, Okamoto K, et al. First report of an Asian family with gastric adenocarcinoma and proximal polyposis of the stomach (GAPPS) revealed with the germline mutation of the APC exon 1B promoter region. Gastric Cancer. 2018;21(6):1058–63. 10.1007/s10120-018-0855-5. 29968043 10.1007/s10120-018-0855-5

[11] Matsumoto C, Iwatsuki M, Iwagami S, Morinaga T, Yamashita K, Nakamura K, et al. Prophylactic laparoscopic total gastrectomy for gastric adenocarcinoma and proximal polyposis of the stomach (GAPPS): the first report in Asia. Gastric Cancer. 2022;25(2):473–8. 10.1007/s10120-021-01253-x. 34554346 10.1007/s10120-021-01253-x

[12] Kanemitsu K, Iwatsuki M, Yamashita K, Komohara Y, Morinaga T, Iwagami S, et al. Two Asian families with gastric adenocarcinoma and proximal polyposis of the stomach successfully treated via laparoscopic total gastrectomy. Clin J Gastroenterol. 2021;14(1):92–7. 10.1007/s12328-020-01290-6. 33242120 10.1007/s12328-020-01290-6

[13] Salami AC, Stone JM, Greenberg RH, Leighton JC, Miick R, Zavala SR, Zeitzer KL, Bakhos CT. Early prophylactic gastrectomy for the management of gastric adenomatous proximal polyposis syndrome (GAPPS). ACS Case Rev Surg. 2022;3(7):62–8. 36909251 PMC9997706

[14] Powers J, Sande CM, Fortuna D, Katona BW. Multifocal intramucosal gastric adenocarcinoma arising in Fundic Gland Polyposis due to gastric adenocarcinoma and proximal polyposis of the stomach. Am J Gastroenterol. 2021;116(1):9. 10.14309/ajg.0000000000000625. 32453049 10.14309/ajg.0000000000000625

[15] Roberts AG, Bujarska M, Bauer M, Brathwaite C, Pelaez L, Reeves-Garcia J. Gastric adenocarcinoma and proximal polyposis of the stomach in a hispanic Pediatric Patient with APC gene variant c.-191T > G. JPGN Rep. 2021;2(4):e123. 10.1097/PG9.0000000000000123. 37206458 10.1097/PG9.0000000000000123 PMC10191560

[16] Iwakawa Y, Yoshikawa K, Okamoto K, Takayama T, Tokunaga T, Nakao T, et al. Four cases of gastric adenocarcinoma and proximal polyposis of the stomach treated by robotic total gastrectomy. Surg Case Rep. 2022;8(1):70. 10.1186/s40792-022-01425-6. 35435526 10.1186/s40792-022-01425-6 PMC9016103

[17] Xicola RM, Li S, Rodriguez N, Reinecke P, Karam R, Speare V, Black MH, LaDuca H, Llor X. Clinical features and cancer risk in families with pathogenic CDH1 variants irrespective of clinical criteria. J Med Genet. 2019;56(12):838–43. 10.1136/jmedgenet-2019-105991. 31296550 10.1136/jmedgenet-2019-105991

[18] Roberts ME, Ranola JMO, Marshall ML, Susswein LR, Graceffo S, Bohnert K, Tsai G, Klein RT, Hruska KS, Shirts BH. Comparison of CDH1 Penetrance estimates in clinically ascertained families vs families ascertained for multiple gastric cancers. JAMA Oncol. 2019;5(9):1325–31. 10.1001/jamaoncol.2019.1208. 31246251 10.1001/jamaoncol.2019.1208 PMC6604087

[19] Howe JR, Mitros FA, Summers RW. The risk of gastrointestinal carcinoma in familial juvenile polyposis. Ann Surg Oncol. 1998;5(8):751–6. 10.1007/bf02303487. 9869523 10.1007/bf02303487

[20] Hizawa K, Iida M, Yao T, Aoyagi K, Fujishima M. Juvenile polyposis of the stomach: clinicopathological features and its malignant potential. J Clin Pathol. 1997;50(9):771–4. 10.1136/jcp.50.9.771. 9389980 10.1136/jcp.50.9.771 PMC500176

[21] Ruiz-Tovar J, Gamallo C. Gastric diffuse hamartomatous polyposis as unique manifestation of peutz-jeghers syndrome. Acta Chir Belg. 2014;114(6):424–6. 26021691 10.1080/00015458.2014.11681057

[22] Møller P, Seppälä TT, Bernstein I, Holinski-Feder E, Sala P, Gareth Evans D, et al. Cancer risk and survival in path_MMR carriers by gene and gender up to 75 years of age: a report from the prospective Lynch Syndrome Database. Gut. 2018;67(7):1306–16. 10.1136/gutjnl-2017-314057. 28754778 10.1136/gutjnl-2017-314057 PMC6031262

[23] Ladigan-Badura S, Vangala DB, Engel C, Bucksch K, Hueneburg R, Perne C, et al. Value of upper gastrointestinal endoscopy for gastric cancer surveillance in patients with Lynch syndrome. Int J Cancer. 2021;148(1):106–14. 10.1002/ijc.33294. 32930401 10.1002/ijc.33294

[24] Sung H, Ferlay J, Siegel RL, Laversanne M, Soerjomataram I, Jemal A, Bray F. Global Cancer statistics 2020: GLOBOCAN estimates of incidence and Mortality Worldwide for 36 cancers in 185 countries. CA Cancer J Clin. 2021;71(3):209–49. 10.3322/caac.21660. 33538338 10.3322/caac.21660

[25] Wagner A, Aretz S, Auranen A, Bruno MJ, Cavestro GM, Crosbie EJ, et al. The management of Peutz-Jeghers Syndrome: European Hereditary Tumour Group (EHTG) Guideline. J Clin Med. 2021;10(3):473. 10.3390/jcm10030473. 33513864 10.3390/jcm10030473 PMC7865862

[26] Syngal S, Brand RE, Church JM, Giardiello FM, Hampel HL, Burt RW. American College of Gastroenterology. ACG clinical guideline: genetic testing and management of hereditary gastrointestinal cancer syndromes. Am J Gastroenterol. 2015;110(2):223–62. 10.1038/ajg.2014.435. quiz 263. 25645574 10.1038/ajg.2014.435 PMC4695986

[27] Jelsig AM, Qvist N, Bertelsen B, Christensen LL, Grossjohan H, Lautrup CK, et al. Distinct gastric phenotype in patients with pathogenic variants in SMAD4: a nationwide cross-sectional study. Endosc Int Open. 2022;10(12):E1537–43. 10.1055/a-1954-0522. 36531685 10.1055/a-1954-0522 PMC9754866

[28] Peacock O, Waters PS, Otero de Pablos J, Boussioutas A, Skandarajah A, Simpson JA, Warrier SK, Heriot AG. A systematic review of risk-reducing cancer surgery outcomes for hereditary cancer syndromes. Eur J Surg Oncol. 2019;45:2241–50. 10.1016/j.ejso.2019.06.034. 31262600 10.1016/j.ejso.2019.06.034

[29] Udredning og opfølgning FAP, A-FAP og GAPPS. V. 1.0. 2021. https://dsmg.dk/kliniske-guidelines/dsmg-guidelines/2021. Accessed 2 Oct 2023.

[30] Heald B, Mester J, Rybicki L, Orloff MS, Burke CA, Eng C. Frequent gastrointestinal polyps and colorectal adenocarcinomas in a prospective series of PTEN mutation carriers. Gastroenterology. 2010;139(6):1927–33. 10.1053/j.gastro.2010.06.061. 20600018 10.1053/j.gastro.2010.06.061 PMC3652614

[31] Correia TF, Mesquita I, Marcos M, Nogueira C, Santos J. Surgical approach to gastric polyposis in Cowen syndrome-case report. J Surg Case Rep. 2021;2021(6):rjab258. 10.1093/jscr/rjab258. 34168854 10.1093/jscr/rjab258 PMC8219395

[32] Kay PS, Soetikno RM, Mindelzun R, Young HS. Diffuse esophageal glycogenic acanthosis: an endoscopic marker of Cowden’s disease. Am J Gastroenterol. 1997;92(6):1038–40. PMID: 9177527. 9177527

[33] Jalving M, Koornstra JJ, Wesseling J, Boezen HM, DE Jong S, Kleibeuker JH. Increased risk of fundic gland polyps during long-term proton pump inhibitor therapy. Aliment Pharmacol Ther. 2006;24(9):1341–8. 10.1111/j.1365-2036.2006.03127.x. 17059515 10.1111/j.1365-2036.2006.03127.x

[34] Islam RS, Patel NC, Lam-Himlin D, Nguyen CC. Gastric polyps: a review of clinical, endoscopic, and histopathologic features and management decisions. Gastroenterol Hepatol (N Y). 2013;9(10):640–51. PMID: 24764778. 24764778 PMC3992058

[35] Wong MCS, Huang J, Huang JLW, Pang TWY, Choi P, Wang J, Chiang JI, Jiang JY. Global prevalence of colorectal neoplasia: a systematic review and Meta-analysis. Clin Gastroenterol Hepatol. 2020;18(3):553–e56110. 10.1016/j.cgh.2019.07.016. 31323383 10.1016/j.cgh.2019.07.016

